# Application of NaP1 Zeolite Modified with Silanes in Bitumen Foaming Process

**DOI:** 10.3390/ma17235902

**Published:** 2024-12-02

**Authors:** Szymon Malinowski, Roman Pacholak, Krzysztof Kołodziej, Agnieszka Woszuk

**Affiliations:** 1 Faculty of Civil Engineering and Architecture, Lublin University of Technology, Nadbystrzycka 40 Street, 20-618 Lublin, Poland; 2 Faculty of Civil Engineering and Environmental Sciences, Bialystok University of Technology, Wiejska 45E Street, 15-351 Bialystok, Poland; roman.pacholak@pb.edu.pl; 3 The Faculty of Civil and Environmental Engineering and Architecture, Rzeszow University of Technology, Powstańców Warszawy 12 Street, 35-959 Rzeszów, Poland; krzych@prz.edu.pl

**Keywords:** zeolite, TEOS, MPTS, TESPT, bitumen foaming

## Abstract

In recent years, global climate change has caused worldwide trends in science and industry toward a focus on the development of modern technologies with reduced environmental impact, including reduced CO_2_ emissions into the atmosphere. The technology for producing asphalt mixtures (AM) at lower temperatures (WMA—warm asphalt mix) using zeolite materials for the bitumen foaming process fits perfectly into these trends. Therefore, towards the development of this technology, the research presented in this paper presents the modification process of zeolite NaP1 from fly ash with silanes of different chemical structures (TEOS, MPTS, TESPT) and their application in the foaming process of bitumen modified with polymers (PMB 45/80-55). The scope of the work includes two main novelty elements: (1) the use of zeolite–silane composites in bitumen foaming and (2) polymer-modified bitumen foaming. Chemical characterisation carried out by EDS-XRF, FTIR, and XPS analysis clearly demonstrated the success of the zeolite matrix modification process, which directly resulted in textural changes. Simultaneously, mineralogical analysis carried out by XRD showed the complete retention of the initial phase composition of zeolite matrix. Further studies have shown that the application of zeolite–oxide composites results in less PMB 45/80-55 stiffening without imposing negative effects on its softening point and dynamic viscosity.

## 1. Introduction

Zeolites are minerals from the aluminosilicate group, characterised by their unique porous structure. Their internal structure consists of a system of chambers and structural channels containing H_2_O molecules. This so-called zeolite water can be removed via temperature increase without destroying the crystal structure of the zeolite. On the other hand, the resulting free pores can be refilled with water molecules or other compounds [1]. These properties are found in both natural and synthetic zeolites. Natural zeolites are formed in sedimentary environments and as a result of hydrothermal transformations. More than 100 different types of this mineral are found in nature. However, only a few are commercially exploited, and the most common are clinoptilolite, chabazite, and mordenite [2].

The growth in zeolite demand has led to the development of technologies for the production of synthetic zeolites with precisely defined structural parameters [3], with simultaneous technology optimisation occurring in terms of its efficiency and energy consumption. The synthesis of zeolites typically involves hydrothermal processes, alkaline fusion, or other innovative methods like microwave heating and sonication, which affect the crystalline structure and chemical composition of the final product. These synthetic methods have enabled the production of zeolites with specific characteristics such as high thermal stability, specific surface area, and cation exchange capacity [1]. To date, more than 200 different zeolite structures have been developed and described, covering not only classical crystalline aluminosilicate materials but also zeolite-like materials with a varied framework chemistry, such as ordered mesoporous materials or porous organic–inorganic materials (e.g., metal–organic frameworks—MOFs) [4]. The diversity of zeolite structures and their physicochemical properties have led to these materials being used in a wide range of industries, such as oil refining, environmental clean-up, construction, and even medicine [5,6,7,8]. Because as part of their structure, zeolites contain zeolite water molecules, which are released at high temperatures, these materials have also found applications in bitumen foaming technology [9], within the warm xix asphalt (WMA) scope. In traditional hot mix asphalt production techniques, bitumen is heated to a temperature of 160–200 °C, which is associated with high emissions of hazardous compounds into the atmosphere and high energy requirements. Due to the use of WMA technology, a reduction in temperature of approx. 20–40 °C is achieved [10], which provides a number of environmental, economic, and environmental benefits. This is possible by:

- introducing additives into the bitumen that reduce its viscosity and improve its adhesion to the aggregate (e.g., waxes, chemical additives)
- foaming the bitumen by adding water, increasing the volume of the bitumen by several times and reducing its viscosity in a short interval.

A combination of water foaming and additive-based WMA technologies are the so-called water carriers, referred to as ‘water-containing technologies’. This group of additives includes zeolites.

When hot bitumen and zeolite water interact, there is an increase in the volume of the so-called bitumen foam, which allows the aggregate grains to be surrounded by the bitumen binder at lower temperatures. In addition, the porous structure of these materials allows them to be activated during the soaking process, which has made it possible to optimise the bitumen foaming process while reducing the amount of zeolite additive used and the cost. Synthetic zeolites with a Linde A-type structure (LTA) produced from chemical reagents were used for the first time in asphalt mixture production technology [11,12], which enabled production temperatures to be reduced by 20 °C. In recent years, WMAs have been extensively investigated using synthetic zeolites produced from waste materials with different structure types: NaP1, NaA, NaX, and sodalite [13]. Highly promising results regarding bitumen properties and asphalt mixtures have also been reported for the use of natural clinoptilolite zeolite [14,15]. Analysis of existing research indicates that the parameters of zeolite materials that directly affect the efficacy of bitumen foaming are the Si/Al ratio in the zeolite backbone, textural parameters, ion-exchange cations type (Na^+^, Ca^2+^), and the amount and binding mechanism of water within the zeolitre crystalline structure [16]. In addition, the cold recycling technology of emulsified bitumen (CRTE) is used to reduce the environmental impact of AM production with, for example, waste materials [17]. An equally important aspect of the sustainable development concept is the extension of the product life cycle. To achieve this, the objective with regard to asphalt mixtures should be to inhibit the bitumen ageing processes of bitumen. It has been observed that an increase in the ageing resistance of bitumen is enabled by the addition of sulphur. In addition, it has been shown that the presence of this element can improve the rheological characteristics of the binder and reduce its fatigue susceptibility [18,19].

**Scheme 1:**
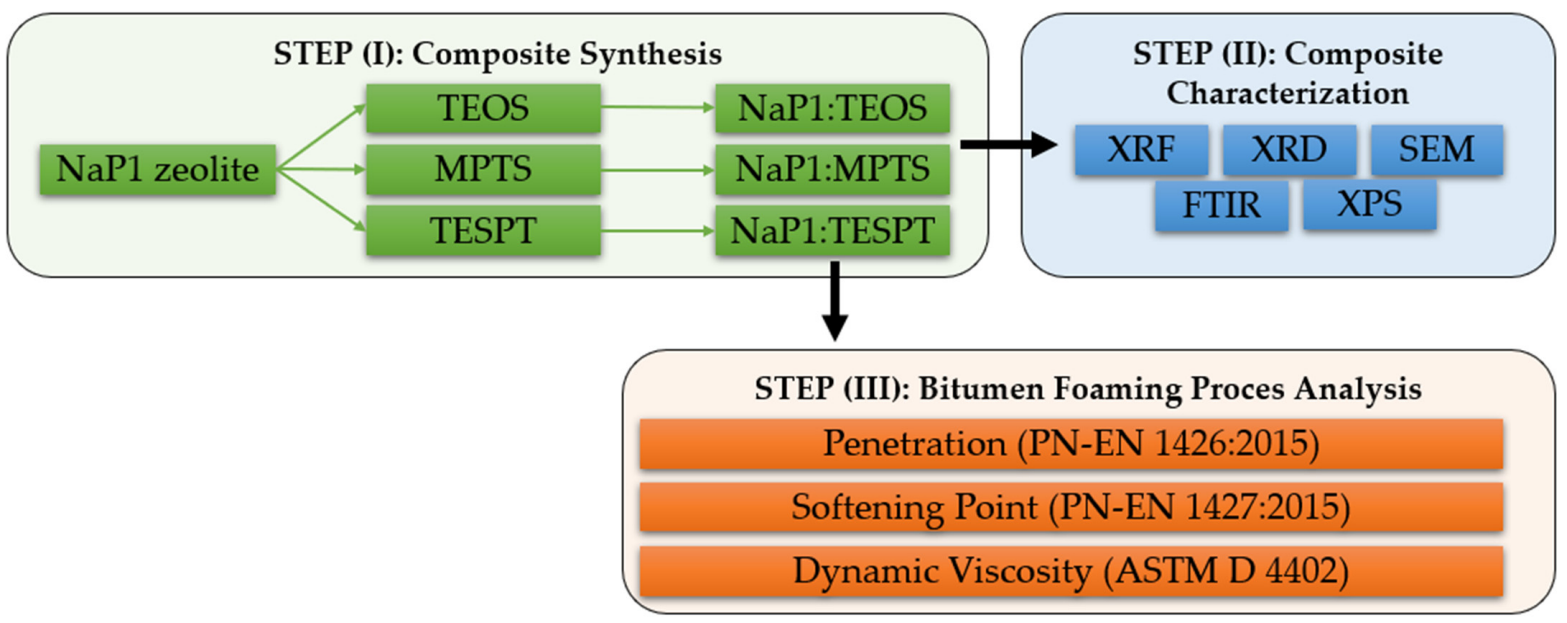
Overall research plan.

Currently, the development of bitumen foaming with zeolite materials includes the process of their modification, which can occur through physical processes and/or chemical reactions. Zeolite modification via physical methods includes the use of high-temperature, microwave, and/or ultrasonic treatment. These treatments aim to evaporate the zeolite water, smooth the channels and chambers, remove impurities from the internal porous structure, and increase the pore diameter and specific surface area [20,21]. Meanwhile, a direct result of zeolite modification by chemical methods is a change in their elemental composition. This effect can be achieved using liquid [22] and solid [23] ion exchange [24], zeolite impregnation with metal precursor solutions [25], or the incorporation of metals into their porous structure [24]. Aluminium atoms present in the zeolite material structure isomorphically substitute silicon atoms, which generates a negative charge in the network, compensated by unbound metal cations of groups I and II of the periodic table, i.e., Na^+^, K^+^, Ca^2+^, Mg^2+^, Sr^2+^, or Ba^2+^. Subsequently, these cations can be easily replaced by ion exchange with, for example, other cations or a proton. To date, cation exchange has been applied to zeolite pore architecture to significantly enhance its structure and consequently its adsorption and diffusion properties [26,27,28,29,30]. As shown in Scheme 1, the zeolite surface is covered with numerous active hydroxyl groups (-OH), which enable it to be effectively modified by chemical methods via the formation of covalent bonds [31]. This methodology has been used for various organic compounds, i.e., APTES (3-Aminopropyltrimethoxysilane) [31], APDEMS ((3-aminopropyl)-diethoxymethyl silane) [32], OTS (octadecyltrichlorosilane) [33], APTMS (aminopropyltrimethoxyxilane) [34], TEOS (tetraethoxysilane) [35], MPTS ((3-Mercaptopropyl)trimethoxysilane) [36], and TESPT (Bis (triethoxysilylpropyl)tetrasulfide) [37].

In line with current trends in the development of bitumen foaming technology, the focus of the research presented in this paper was the modification of synthetic zeolite obtained by fly ash hydrothermal conversion and the application of the obtained composites in the foaming of polymer-modified road bitumen (PMB). To date, zeolite–silane composites have not been used in the foaming of road bitumen in the literature. However, a detailed analysis of the silane chemical structure used in the modification of zeolite matrices indicates that they can effectively increase the number of active binding sites for water molecules during their activation, which will directly result in an increase in the efficiency of the foaming process. In order to verify the above hypothesis, a series of research steps were undertaken, shown graphically in Scheme 1.

## 2. Materials and Methods

### 2.1. Materials

**Figure 1:**
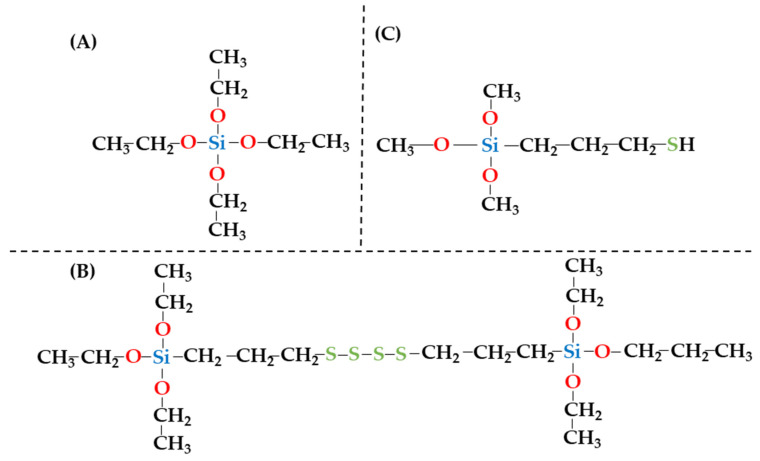
Chemical structure of TEOS (**A**), MPTS (**B**), and TESPT (**C**)**.**

For the synthesis of the composite materials, organic compounds from the silane group with different chemical structures, as shown in Figure 1, were used, i.e., TEOS (tetraethoxysilane), MPTS ((3-Mercaptopropyl)trimethoxysilane), and TESPT (Bis (triethoxysilylpropyl)tetrasulfide). All of these were obtained from Sigma Aldrich (branch Poznań, Poland) and used in composite synthesis without any purification. During the modification process, suitable organic solvents were also required, i.e., ethanol (C_2_H_5_OH), acetic acid (CH_3_COOH), and toluene (C_7_H_8_), which were obtained from Chempur (Piekary Śląskie, Poland) and used without purification.

### 2.2. NaP1 Zeolite Matrix Synthesis

The zeolite matrix of structure type NaP1 obtained by conversion fly ash was used to synthesise the composites investigated in this paper. According to the currently used nomenclature, zeolite NaP1 represents a gismondine-like framework (GIS), where 8-membered channels of 3.1 × 4.5 Å and 2.8 × 4.8 Å in diameter are formed of 2 4-membered aluminosilicate rings. The synthesis was performed in a semi-technical reactor using 20 kg of fly ash, 12 kg of sodium hydroxide (NaOH), and 90 dm^3^ of water to obtain a matrix with a well-defined pore structure. The conversion reaction was carried out at T = 80 °C for 36 h [3,38]. The zeolite matrix obtained in this way was used in the modification process presented in detail in Section 2.2.

### 2.3. Composite Synthesis

Subsequently, zeolite matrices obtained from fly ash according to the procedure presented in Section 2.2 were used to synthesise NaP1:TEOS, NaP1:MPTS, and NaP1:TESPT composites. To obtain the NaP1:TEOS composite, 6.0 g of zeolite matrix was weighed out and 50 mL of C2H5OH was added at a concentration of 95% *v*/*v*. The pH was then set at 3.5 using CH3COOH, and 0.09 g of TEOS was added. The prepared mixture was hydrolysed at room temperature for 20 min, and then the temperature was increased to 70 °C and grafting was carried out for 2 h. The resulting composite was washed with water and C2H5OH and dried at T = 60 °C for 24 h [35]. To obtain the NaP1:MPTS composite, 15.0 g of zeolite matrix was weighed out into a beaker, and 30 mL of toluene and 0.225 g of MPTS were added. The mixture was then stirred at T = 60 °C for 6 h, and the resulting composite was washed with toluene [36]. Meanwhile, to obtain the NaP1:TESPT composite, 30 g of zeolite matrix, 60 mL of acidified toluene (pH = 3.5–4.5), and 0.450 g of TESPT were placed in a beaker. Thereafter, the above mixture was stirred for 4 h at T = 80 °C, and the obtained composite was washed with water until a filtrate of pH = 7 was obtained [37].

### 2.4. Testing Methods

The NaP1 zeolite and zeolite–silane composite obtained by the procedure detailed in Section 2.3 were then characterised in terms of chemical and mineralogical composition. The ED-XRF method (energy dispersive X-ray fluorescence spectroscopy, using an Epsilon 3x spectrometer, Malvern Panalytical, Malvern, UK) was used to analyse the chemical composition. Meanwhile, FTIR (Fourier-transform infrared spectroscopy) and XPS (X-ray photoelectron spectroscopy) techniques were used to analyse the chemical structure of the zeolite matrix and zeolite–silane composites. FTIR spectra were recorded with a spectrometer NICOLET 380 (Thermo Scientific, Waltham, MA, USA) in the wavenumber range 4000–400 cm^−1^ at a resolution of 4 cm^−1^. The recorded interferograms consisted of 1024 scans, which guaranteed an appropriate signal-to-noise ratio and allowed their analysis without additional smoothing. XPS analysis of NaP1, NaP1:TEOS, NaP1:MPTS, and NaP1:TESPT was carried out in high vacuum using a multi-chamber UHV system from Prevac equipped with an excitation radiation source in the form of an aluminium anode emitting monochromatic radiation, with an AlKα characteristic line and an energy of 1486.6 eV. The mineralogical composition of the tested porous materials was analysed by X-ray diffraction XRD (Panalytical, Almelo, The Netherlands). XRD patterns were analysed via the powder method using a Panalytical X’pert PROMPD X-ray diffractometer with a PW 3050/60 goniometer in the angle range 5–65 2θ equipped with a Cu copper lamp (CuKα = 0.154178 nm) as the source of X-radiation. The data obtained were then processed using X’ Pert High Score software (ver. 5.1). Mineral phases identification was based on a PDF-2 (released 2010) database and formalised by JCPDS/ICDD. A Quanta 250 FEG by FEI (Hilsboro, OR, USA) scanning electron microscope (SEM) was used to analyse the morphology of the zeolite matrix and the resulting zeolite–silane composites, and textural properties were analysed based on low-temperature nitrogen adsorption/desorption isotherm, determined using a Micromeritics ASAP 2020 instrument (Norcross, GA, USA).

After detailed characterisation, the porous materials were used in the foaming process of commercially available polymer-modified bitumen PMB 45/80-55, produced by Orlen Asphalt S.A, which had previously been liquefied at 160 °C for 2 h. The binder prepared in this way was weighed out into a metal can and then a weight of NaP1, NaP1:TEOS, NaP1:MPTS, or NaP1:TESPT of 5% *w*/*w* was prepared in a separate vessel in relation to the weight of the previously weighed binder. The weighed porous material was then activated with water at 75% *w*/*w* relative to its weight and added to the liquefied PMB 45/80-55 bitumen. For NaP1, NaP1:TEOS, and NaP1:MPTS foamed bitumen, penetration was determined according to PN-EN 1426:2009 at 25 °C using a semi-automatic penetrometer. Meanwhile, the softening point of the obtained foamed bitumen binders was determined using the ‘ring and ball’ method according to PN-EN 1427:2009. In order to measure the dynamic viscosity of the foamed bitumen binders, a Brookfield viscosity meter was used, and the measurement was carried out at T = 135 °C at 3 time intervals: 15, 30, and 45 min.

## 3. Results and Discussion

### 3.1. Characterisation of Zeolite–Silane Composites

Firstly, the chemical composition of the zeolite–silane composites studied was analysed. In terms of the modification process of the zeolite matrices, the most important contents are the percentages of Al_2_O_3_, SiO_2_, and SO_3_. Table 1 clearly shows that the modification process of the NaP1 zeolite matrix with TEOS and MPTS does not significantly affect the Al_2_O_3_ content. For the unmodified zeolite matrix, the content of this component was determined to be 17.76%, while for the NaP1:TEOS and NaP1:MPTS composites, this increased slightly to 18.61% and 17.97%, respectively. Meanwhile, for the NaP1:TESPT composite, a slightly lower Al_2_O_3_ content of 14.49% was determined. This means that in a toluene solution of pH = 3.5–4.5, modification causes leaching of Al_2_O_3_ from the zeolite matrix. Table 1 also indicates that the modification process of the zeolite matrix with TEOS, MPTS, and TESPT results in an increase in SiO_2_ content of about 3% compared to the unmodified NaP1 zeolite matrix. These changes confirm that the modification process of the NaP1 zeolite matrix was successful and the TEOS, MPTS, and TESPT molecules were effectively grafted to the aluminosilicate backbone. Considering the chemical structure of the silanes used in the modification process, it is also very important to analyse the sulphur content of the composites obtained. Table 1 shows that the NaP1:MPTS and NaP1:TESPT composites were characterised by increased SO_3_ content. The observed increase was 2.37% and 8.30%, respectively. Considering the chemical structures of the silanes used in the modification of the zeolite matrices, these changes also confirm the successfulness of the NaP1 zeolite matrix modification process. Equally important is the fact that a noticeably higher SO_3_ content was determined for the NaP1:TESPT composite, which is a direct result of the presence of four S atoms in the chemical structure of TESPT (Figure 1C).

**Figure 2:**
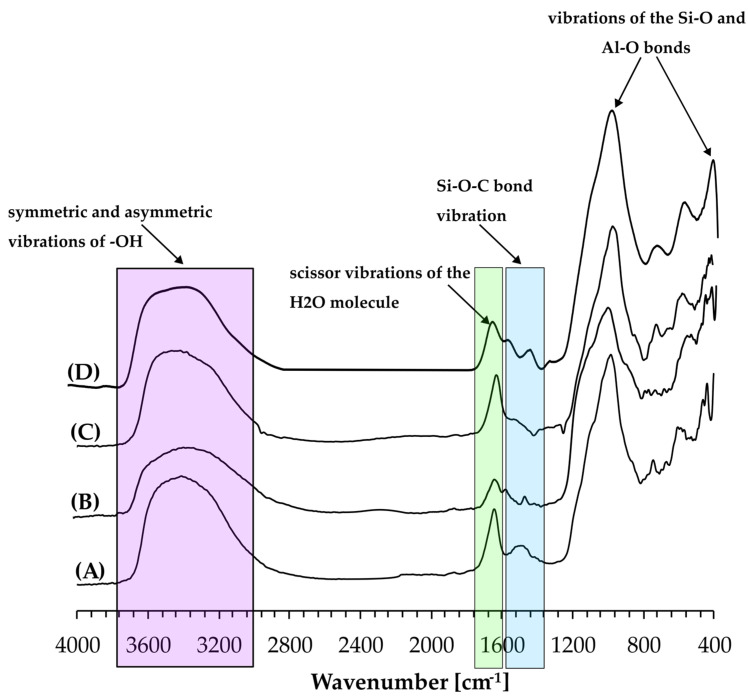
FTIR spectra of NaP1 zeolite matrix (**A**), as well as NaP1:TEOS (**B**), NaP1:MPTS (**C**), and NaP1:TESPT (**D**) composites.

The chemical structure of the NaP1 zeolite matrix and the NaP1:TEOS, NaP1:MPTS, and NaP1:TESPT composites was also investigated by FTIR analysis. Figure 2A–D shows that the spectra of all the analysed materials include a wide band at a wavenumber of approximately 3400 cm^−1^. This derives from the symmetric and asymmetric vibrations of -OH groups of the H_2_O molecules adsorbed on the surface of the zeolite matrix. The presence of these vibrations is also confirmed by a band located at a wavenumber of approximately 1630 cm^−1^, attributed to the scissor vibrations of the H_2_O molecule [39,40]. The FTIR spectra seen in Figure 2A–D also contain bands derived from the vibrations of aluminosilicates. The bands at approximately 460 cm^−1^ and 1000 cm^−1^ correspond to the vibrations of the Si-O and Al-O bonds that build up the aluminosilicate tetrahedrons of the zeolite matrix, and the bands at approximately 670 cm^−1^ and 740 cm^−1^ correspond to the double-ring external linkage vibrations and symmetric stretch vibrations of internal tetrahedrons, respectively. Besides these typical bands characteristic of a NaP1-type zeolite matrix, the spectra of the NaP1:TEOS, NaP1:MPTS, and NaP1:TESPT composites (Figure 2B–D) show additional bands indicating the presence of organic compounds covalently attached during the modification. In the spectra of the composites, a band with a wavenumber of approximately 1400 cm^−1^ corresponding to Si-O-C [41] bond vibrations was observed, which directly confirms the successful modification process of the zeolite matrix.

**Figure 3:**
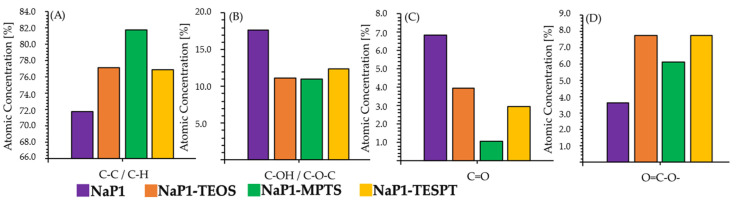
Atomic concentration of carbon in the form of C-C/C-H (**A**), C-OH/C-O-C (**B**), C=O (**C**), and O=C-O-, (**D**) on NaP1, NaP1:TEOS, NaP1:MPTS, and NaP1:TESPT.

The surface chemistry of the NaP1 zeolite matrix and the investigated zeolite–silane composites was examined by XPS, which showed the presence of C coming from organic impurities occurring as C-C/C-H, C-OH/C-OC, C=O, and O=C-O-. In general, the surface analysis carried out showed an increase in the total content of carbon atoms due to the modification process of the zeolite matrix, with organic compounds belonging to the silane group. On the surface of the unmodified zeolite matrix, a total carbon content of 10.2% was determined, while on the surface of the NaP1:TEOS, NaP1:MPTS, and NaP1:TESPT composites, the total carbon content was determined to be 18.4%, 25.4%, and 18.5%, respectively. This clearly indicates that zeolite matrix modification with MPTS is most effective on the surface of the zeolite matrix. The identified carbon on NaP1 zeolite matrix and investigated composites occurs in different forms, whose content on the surface of the unmodified zeolite matrix increases as follows: C-C/C-H>C-OH/C-O-C>C=O>O=C-O-. The surface analysis of the organic–mineral composites studied shows that the content of C-C/C-H bonds on the surface increases as a result of the NaP1 zeolite matrix modification process. For the unmodified zeolite matrix, their content was determined to be approximately 71.8%, while for the NaP1:TEOS, NaP1:MPTS, and NaP1:TESPT composite, the content of C-C and C-H bonds was 77.8%, 81.8%, and 76.9%, respectively. Similarly increasing trends were observed for C occurring in the form O=C-O-. Carbon atoms in this form accounted for 3.6% of the surface area of the unmodified NaP1 zeolite matrix, while after the chemical attachment of TEOS, MPTS, and TESPT molecules, their surface content increased to 7.7%, 6.2%, and 7.7%, respectively. In contrast, exactly opposite trends were observed for carbon atoms in the form of C-OH/C-O-C and C=O. In the first case, the surface content decreased from 17.7% for the unmodified NaP1 zeolite matrix to 11.1% for the NaP1:TEOS composite, 11.0% for the NaP1:MPTS composite, and 12.4% for the NaP1:TESPT composite. Meanwhile, as can be seen in Figure 3, the content of C=O groups on the post-modified NaP1 zeolite had decreased by 2.9% after modification with TEOS, 5.8% after modification with MPTS, and 3.9% after modification with TESPT.

XPS analysis was used to determine the content of O chemically bonded to Si atoms in the form of Si-O on the surface of the NaP1 zeolite matrix and composites obtained from its modification. These bonds are present both in the aluminosilicate framework of the zeolite matrix and in the structures of the silanes used in the modification shown in Figure 1. Analysis of the chemical composition of the zeolite matrix surface showed that the O content in the form of Si-O bonds was 34.6%. Meanwhile, the atomic concentrations of Si-O bonds on the surface of NaP1:TEOS, NaP1:MPTS, and NaP1:TESPT composites were determined to be 44.0%, 41.7% and 47.2%, respectively. This clearly indicates that the modification process of the NaP1 zeolite was successful, and the organic molecules TEOS, MPTS, and TESPT were chemically attached to the zeolite. In addition, XPS surface analysis also revealed the presence of oxygen present in the Si-OH form. However, in this case, no effect of the modification on its surface content was observed. For NaP1, NaP1:TEOS, NaP1:MPTS, and NaP1:TESPT, very comparable percentages of the Si-OH group ranging from 3.0% to 4.0% were determined.

**Figure 4:**
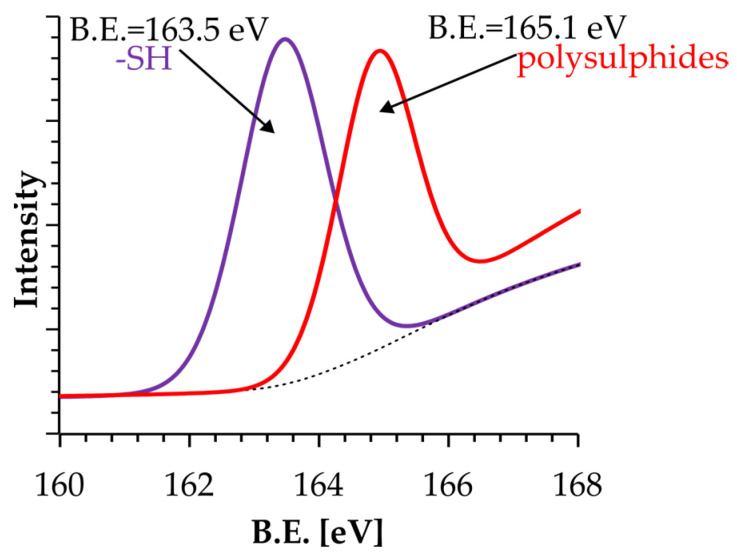
High-resolution XPS of S 2p spectra of NaP1:MPTS and NaP1:TESPT composites.

From the point of view of the modification of the zeolite matrix with silanes, the identification of S atoms is also critical. As shown in Figure 1, S atoms occur in the -SH form within the MPTS molecule and in the -S-S-S-S- form within the TESPT molecule. Therefore, validation of the modification process of the zeolite matrix can also be confirmed from analysis of the S forms that occur. Neither on the surface of the unmodified zeolite NaP1 nor on the NaP1:TEOS composite was the presence of S identified, which is due to the chemical structure of both the zeolite NaP1 and the TEOS molecule. However, meaningfully, the presence of S atoms was identified for the NaP1:MPTS and NaP1:TESPT composites. As can be seen in Figure 4, S is present in these composites in the form of -SH and -S-S-S-S- groups, respectively, which is in complete accordance with the chemical structures of the MPTS and TESPT silane shown in Figure 1B,C.

**Figure 5:**
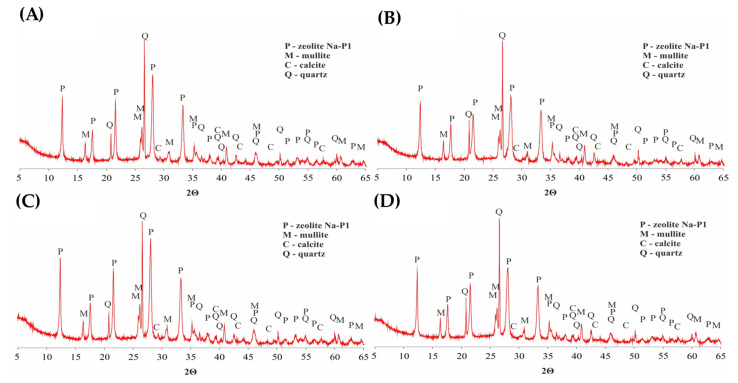
XRD patterns of NaP1 zeolite (**A**), NaP1:TEOS (**B**), NaP1:MPTS (**C**), and NaP1:TESPT (**D**) composite.

The mineralogical composition of the zeolite matrix and the zeolite–silane composites studied was then analysed. Figure 5A clearly indicates the presence of a zeolite phase of the NaP1 structure type. This is indicated by reflections corresponding to d_hkl_ values of 2.68, 3.17, 4.10, 5.02, and 7.10 Å. Mineralogical analysis also revealed the presence of mullite in the zeolite matrix, which was indicated by diffraction peaks corresponding to d_hkl_ values of 1.52, 2.54, 3.40, 3.42, and 5.40 Å, whereas peaks corresponding to d_hkl_ values of 1.37, 1.54, 1.81, 3.34, and 4.26 Å indicate the presence of quartz in the zeolite matrix as well. A comparison of the XRD patterns recorded for the NaP1 zeolite (Figure 5A) and the composites obtained based on zeolite (Figure 5B–D) clearly indicates that the mineralogical composition of the matrix is not affected by the modification process. All recorded XRD patterns indicate the presence of NaP1 zeolite, mullite, and quartz. As outlined in Section 2.2, the zeolite–silane composites studied were obtained under non-aqueous conditions. The use of such conditions for the silane modification of other porous materials has also made it possible to preserve the original mineralogical structure. Such an effect has been observed for SBA-3 [42], zeolite W [40], natural zeolite [34], and NaA [33]. Typically, a characteristic elevation of the background in the range 2Θ 10–35 [43] is observed in zeolite–organic composites. However, due to the relatively small amount of grafeted silanes, this effect was not observed for the investigated composites.

**Figure 6:**
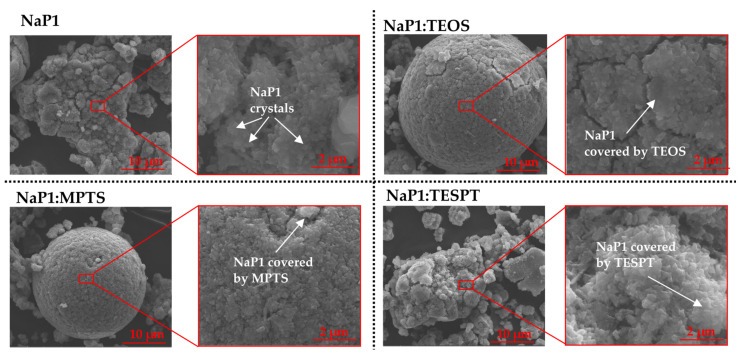
SEM images of NaP1 zeolite, as well as NaP1:TEOS, NaP1:MPTS, and NaP1:TESPT, at magnifications of 8000 and 60,000.

Additionally, the morphological analysis of the NaP1 zeolite matrix and investigated composites was carried out using a scanning electron microscope (SEM). The analysis shows that the NaP1 zeolite matrix synthesised from fly ash forms small plates with irregular shapes forming clusters of different sizes (Figure 6). In contrast, SEM analysis of the NaP1:TEOS, NaP1:MPTS, and NaP1:TESPT composites indicates that, as a result of the modification process, the zeolite crystals are coated with amorphous organic matter. Similar observations are presented in [43] for the β-cyclodextrin-modified NaX zeolite and in [44] for the chitosan-modified NaP1 zeolite.

**Figure 7:**
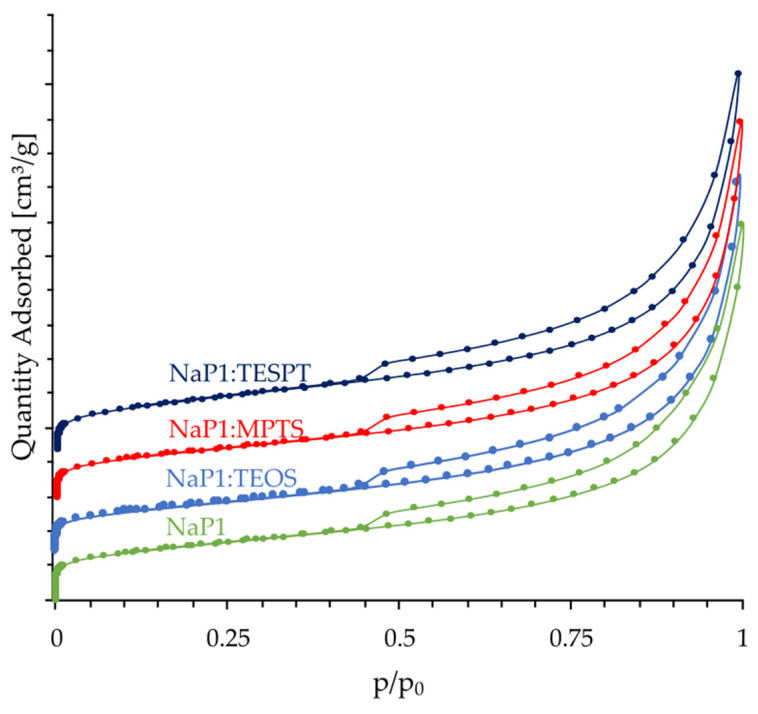
Nitrogen adsorption/desorption isotherms of the NaP1 zeolite matrix, as well as NaP1:TEOS, NaP1:MPTS, and NaP1:TESPT composites.

Figure 7 shows the nitrogen adsorption/desorption isotherms of the NaP1 zeolite matrix and the composites NaP1:TEOS, NaP1:MPTS, and NaP1:TESPT. The shape and hysteresis loop clearly indicate that the porous structure of the zeolite matrix used for the synthesis of zeolite–silane composites is composed of both micro and mesopores. Furthermore, as can be seen in Figure 7, the shape and hysteresis loop determined for the NaP1:TEOS, NaP1:MPTS, and NaP1:TESPT composites show that the chemical modification process of the NaP1 zeolite does not affect its three-dimensional pore structure. However, further texture analysis of the zeolite matrix and the investigated composites indicates that chemical grafting of organic compounds from the silane group to the aluminosilicate skeleton affects the BET specific surface area. For the NaP1 zeolite matrix, the determined value was 73 m^2^/g, while after its modification with TEOS, MPTS, and TESPT, this decreased to values of 62 m^2^/g, 57 m^2^/g, and 63 m^2^/g, respectively. Compared to the BET specific surface area values determined for pure zeolite NaP1 matrix, these values are lower by 15.4%, 21.8%, and 14.3%, respectively. Such an effect has also been observed as a result of silane modification being applied to other porous materials. Chen et al. [42] observed a reduction in the BET specific surface area of SBA-3 by approximately 24%, with an original SBA-3:TEOS ratio of 1.1/0.5. In contrast, Abdellaoui et al. [40] observed that the modification of zeolite W with APTES resulted in a decrease in BET specific surface area from 9.67 m^2^/g to 1.40 m^2^/g. Interesting correlations were also observed for external and micropore surface, which decreased after the modification process. The external surface of the NaP1:TEOS, NaP1:MPTS, and NaP1:TESPT composites were lower by 7.2%, 8.3%, and 1.83%, respectively. Meanwhile, the micropore surface area of the NaP1 zeolite decreased by 60.4%, 94.7%, and 82.3%, respectively. The values clearly indicate that during the modification, silanes are attached to the zeolite matrix mainly inside the zeolite matrix micropores of the NaP1 structure type. Similar changes were explained by Sanaeepur et al. [45] based on the blocking and narrowing of zeolite pores.

### 3.2. Properties of Bitumen Foamed with Zeolite–Silane Composites

The dynamic viscosity of bitumen is an important parameter determining the production and compaction temperature of asphalt mixtures. The liquefaction of bitumen with an increase in temperature is necessary to achieve a sufficiently low viscosity, allowing accurate coating of the aggregate grains with a binder film of the required thickness. The viscosity of bitumen decreases with increasing temperature, but the dependence of bitumen viscosity on its temperature is logarithmic, and even small changes in temperature cause large changes in viscosity. In general, when unmodified road bitumen is applied, the binder should be heated to a temperature at which a viscosity of around 0.2 Pa∙s can be reached [46]. This range is achieved when normal road bitumen is heated to temperatures of around 150–195 °C, depending on the bitumen type and hardness. Also, the final compaction temperature of the AM is dependent on the viscosity of the bitumen [47]. However, in the case of modified bitumen, referred to as high-viscosity bitumen, the above-mentioned viscosity limits are not applicable in the determination of AM process temperatures [48].

In a preliminary study on the effect of silane-modified zeolites on the properties of foamed PMB 45/80-55 bitumen with a viscosity of 1435 mPa s measured at 135 °C, it was observed that this value remained at a similar level during subsequent measurements after 30 and 45 min, decreasing by only 5 mPa∙s (Table 2). In bitumen foamed with unmodified NaP1 zeolite, a viscosity of 1500 mPa∙s was obtained for the first measurement (after 15 min), which decreased proportionally with time, reaching 1475 mPa∙s after 45 min. After the application of the NaP1-TEOS composite, a bitumen viscosity higher than that of the reference sample of 1460 mPa∙s was also obtained after a test duration of 45 min. When the bitumen was foamed with NaP1:TESPT composite, the viscosity result was the same as for the unfoamed PMB 45/80-55 bitumen. In contrast, when the zeolite NaP1:MPTS composite was applied, a slight decrease in viscosity was obtained at 45 min compared to the reference bitumen. Summarising, it can be concluded that the zeolite modification methods used have no significant effect on the dynamic viscosity of the modified bitumen. It is worth noting that zeolites are not used as an asphalt modifier, but only as a ‘water carrier’ to foam the bitumen. The lowering of the production temperature is due to the fact that the bitumen foam has a large volume which allows it to surround the aggregate grains at lower temperatures. After the foaming process is complete, the zeolite remains as an undissolved solid in the bitumen, acting as a filler, which increases the viscosity of the bitumen binder [49]. It is also noteworthy that the viscosity measurement is carried out after the foaming process has stopped. Previous studies have determined the effect of zeolite addition on the dynamic viscosity of ordinary road bitumen. A research team led by Sengoz [14,15] observed that using a 5% addition of natural zeolite and commercial Aspa-Min could reduce the dynamic viscosity of bitumen. However, the results obtained by Akisetty et al. [50,51] indicate that an increase in dynamic viscosity is obtained after the application of Aspa-Min zeolite. A similar effect was observed for Linde-A-type (Advera) zeolite [52]. In a study on the effect of foaming bitumen with zeolites of different structural types, Woszuk et al. observed that, regardless of the zeolite type, a lower bitumen viscosity can be obtained when the materials are additionally activated by soaking in water [9] or oil additives [53].

As can be seen in Table 2, the penetration of the PMB 45/80-55 bitumen was 65.8 × 0.1 mm. In the case of bitumen foamed with zeolite materials, a slight stiffening is apparent due to the introduction of a non-dissolved solid into the bitumen matrix. With unmodified NaP1 zeolite matrix, the greatest decrease in penetration of 5% was obtained, to a level of 62.5 × 0.1 mm. In contrast, for bitumen samples foamed with zeolites modified with silanes, a slightly lower decrease in penetration was obtained, ranging from 3.4% to 3.9%. The measured penetration values indicate that the degree of foamed bitumen stiffening is not affected by the type or chemical structure of the silane used in the synthesis of the composite when the procedure outlined in Section 2.2 is used.

The softening point of the PMB 45/80-55 bitumen was 59.5 °C. The application of NaP1 zeolite matrix and NaP1:TEOS, NaP1:MPTS, and NaP1:TESPT composites resulted in a slight decrease in the parameter tested to 59.2–59.8 °C. The difference between the results depending on the type and chemical composition of applied porous material was only 0.6 °C and was within the measurement error. It can therefore be concluded that the zeolite modification with silanes had no effect on the softening temperature of polymer-modified bitumen compared to the results obtained for reference asphalt.

The preliminary results obtained for the influence of zeolite modification on the properties of foamed bitumen showed no significant changes in dynamic viscosity, penetration, or softening temperature. However, it has been confirmed that dynamic viscosity tests are not sufficient to assess the suitability of zeolites for bitumen foaming. In analysing the influence of zeolite properties on the foaming effect of bitumen, Woszuk et al. pointed out that the amount of zeolite water and the way it is released in the temperature range of asphalt mixture production are important parameters. Another important criterion for the bitumen foaming process is the Si/Al ratio in the zeolite structure. A higher ratio results in the zeolite possessing a higher water-binding strength, which results in slower water release. Meanwhile, properties such as mesopores, specific surface area, and volume determine a higher number of water binding sites per unit mass, which translates to a greater amount of water that can be introduced into the zeolite structure [16]. This allows greater amounts of water to be incorporated into the zeolite structure, resulting in a larger volume of bitumen foam.

## 4. Conclusions

In an age of increasing environmental awareness in society, it is necessary to develop environmentally friendly technologies that can be successfully applied to road construction. In response to these challenges, pioneering research has been carried out into the possibility of using mineral–organic composites obtained by the chemical attachment of silanes—i.e., TEOS, MPTS and TESPT—to a NaP1 zeolite matrix. This study showed that it is possible to efficiently obtain zeolite–oxide composites. The presence of organic matter on the surface of the mineral matrix was demonstrated both chemically by XRF, FTIR, and XPS analysis and with regard to morphological changes by SEM microphotography. Investigations carried out in terms of the physicochemical properties of the PMB 45/80-55-modified bitumen observed that the use of zeolites leads to its stiffening. However, this effect was effectively reduced using the foaming process studied here, which was carried out with the zeolite–oxide composites. Furthermore, the study also showed that these composites’ introduction into the bitumen matrix does not negatively affect its physicochemical properties. In order to fully explore the potential of the prepared zeolite–silane composites and to optimise the modification method of zeolites for use in bitumen foaming processes, further studies are required, including:

(1) evaluation of the effect of introducing a higher amount of silane into the zeolite matrix, which should have a positive effect on the change in surface energy of the foamed bitumen;
(2) evaluation of the rheological properties and testing of the surface free energy of bitumen foamed with zeolite–silane composites.

The findings attained so far indicate that zeolite–silane composites can be successfully used in road construction engineering in the production of asphalt mixtures using foamed bitumen technology, which is becoming more frequently employed by road companies. The research conducted represents an important step in the development of bitumen binder foaming technology, as it uses composite materials for the first time. To date, similar research has only been carried out with unmodified zeolite matrices.

## Figures and Tables

**Table 1:** Percentage content of selected components in zeolite matrices and investigated zeolite–silane composites.

	NaP1	NaP1:TEOS	NaP1:TESPT	NaP1:MPTS
Al_2_O_3_	17.76	18.61	17.97	14.49
SiO_2_	33.43	37.14	37.33	37.46
SO_3_	0.14	0.09	2.52	8.44

**Table 2:** Viscosity, penetration, and softening point of PMB 45/80-55 foamed with NaP1 zeolite, as well as that foamed with NaP1:TEOS, NaP1:MPTS, or NaP1:TESPT composites.

	Viscosity at 135 °C			Penetration [0.1 mm]	Softening Point [°C]
Time [min]	15	30	45	X	X
PMB asphalt	1435	1435	1430	65.8	59.5
PMB + NaP1	1500	1480	1475	62.5	59.2
PMB + NaP1-TEOS	1485	1465	1460	63.2	59.6
PMB + NaP1-MPTS	1435	1420	1410	63.6	59.8
PMB + NaP1-TESPT	1435	1435	1430	63.4	59.3

## Data Availability

The original contributions presented in this study are included in the article. Further inquiries can be directed to the corresponding authors.
